# Cell-repellent polyampholyte for conformal coating on microstructures

**DOI:** 10.1038/s41598-022-15177-8

**Published:** 2022-06-25

**Authors:** Kohei Suzuki, Yoshiomi Hiroi, Natsuki Abe-Fukasawa, Taito Nishino, Takeaki Shouji, Junko Katayama, Tatsuto Kageyama, Junji Fukuda

**Affiliations:** 1 Faculty of Engineering, Yokohama National University, 79-5 Tokiwadai, Hodogaya-ku, Yokohama, Kanagawa 240-8501 Japan; 2 Nissan Chemical Corporation, 2-5-1 Nihonbashi, Chuo-ku, Tokyo, 103-6119 Japan; 3 Kanagawa Institute of Industrial Science and Technology, 3-2-1 Sakado Takatsu-ku, Kawasaki, Kanagawa 213-0012 Japan

**Keywords:** Biochemistry, Biotechnology, Medical research, Chemistry, Materials science

## Abstract

Repellent coatings are critical for the development of biomedical and analytical devices to prevent nonspecific protein and cell adhesion. In this study, prevelex (polyampholytes containing phosphate and amine units) was synthesized for the fine coating of microdevices for cell culture. The dip-coating of the prevelex on hydrophobic substrates altered their surfaces to be highly hydrophilic and electrically neutral. The range of prebake temperature (50–150 °C) after dip-coating was moderate and within a preferable range to treat typical materials for cell culture such as polystyrene and polydimethylsiloxane. Scanning electron microscopy revealed a conformal and ultra-thin film coating on the micro/nano structures. When compared with poly(2-hydroxyethyl methacrylate) and poly(2-methacryloyloxyethyl phosphorylcholine), prevelex exhibited better characteristics for coating on microwell array devices, thereby facilitating the formation of spheroids with uniform diameters using various cell types. Furthermore, to examine cellular functionalities, mouse embryonic epithelial and mesenchymal cells were seeded in a prevelex-coated microwell array device. The two types of cells formed hair follicle germ-like aggregates in the device. The aggregates were then transplanted to generate de novo hair follicles in nude mice. The coating material provided a robust and fine coating approach for the preparation of non-fouling surfaces for tissue engineering and biomedical applications.

## Introduction

Nonspecific adhesion of proteins and cells to substrates is considered as an important challenge in biomaterial science fields involving medical implants, biosensors, and cell culture devices^1^. Biofouling may cause adverse inflammatory responses on implants^2^, compromise detection signals on biosensors^3^, and result in random and uncontrolled adhesion of cells on culture devices^4,5^. To solve these problems, intensive research was performed to examine coating materials using various synthetic and natural polymers^6^. A cell-repellent coating on microdevice surfaces is used in the tissue engineering and regenerative medicine fields to induce and maintain cell aggregation for spheroid and organoid culture^7^.

It is generally accepted that the nonfouling properties of a material are closely associated with the hydration and surface charge of materials^8^. A non-fouling material should be neutral in charge and contain hydrophilic polar functional groups^9^. There are two types of nonfouling materials: nonionic and zwitterionic. Poly(ethylene glycol) (PEG) is a typical nonionic hydrophilic material^1^. Specifically, PEG is proven to be nonfouling, nontoxic, and nonimmunogenic. Other typical nonionic materials include 2-hydroxyethyl methacrylamide (pHEMA)^10,11^ and *N*-(2-hydroxypropy) methacrylamide^12,13^. These polymers are used in a wide range of biomedical applications such as implants, biosensors, and tissue engineering applications^10,14^. Zwitterionic polymers are categorized into two main types: polybetaines and polyampholytes^15^. Polybetaines contain positively and negatively charged species on the same monomer, whereas polyampholytes contain those on different co-polymers. Zwitterionic materials exhibit significantly stronger hydration effects and better antifouling properties than nonionic materials, potentially caused by the ionic species^16^. Typical polybetaines are poly(sulfobetaine methacrylate), poly(carboxybetaine methacrylate), and polymers with 2-methacryloyloxyethyl phosphorylcholine (MPC polymers)^17–20^. Typical combinations of copolymers of polyampholytes are composed of [2-(methacryloyloxy)ethyl]trimethylammonium chloride and [2-(acryloyloxy) ethyl] trimethyl ammonium chloride for the positive charge, and 3-sulfopropyl methacrylate potassium salt and 2-carboxyethyl acrylate for the negative charge^21–23^. Among zwitterionic polymers, MPC polymers contain a phospholipid polar group that mimics a cell membrane and are completely harmless to cells, and extensive investigations were conducted on antifouling coating to biomedical devices and even oral cavity including clinical trials^24^.

In addition to the intrinsic nonfouling chemical nature, several other factors play important roles in determining the antifouling performance and applicability^25^. These include conformal and homogeneous coating, gamma sterilization, stability in stock and biological environments, biocompatibility, and costs.

Conformal and homogeneous coatings are fundamental for the biomedical applications, especially for micro/nanostructures. In cell culture, micro/nano fabrication approaches such as photolithography were adapted to engineer microwell arrays, body on a chip devices, and finely-controlled scaffolds for drug testing and tissue engineering applications^26–29^. However, uniform and ultrathin coating on micro/nanostructures is particularly challenging^30^. Ultrathin coatings were investigated using various approaches including surface initiated-atom transfer radical polymerization (SI-ATRP), self-assembled monolayer (SAM), and vapor deposition^31–33^. Specifically, ATRP is used to fabricate dense and uniform polymer brushes composed of nonionic hydrophilic units or zwitterionic units with ultrathin thickness (~ 30 nm). Alkanethiol SAMs terminated with oligoethylene glycol were used to prevent protein adsorption. Vapor deposition constitutes another approach for coating a surface with an ultrathin polymer layer. Surfaces modified with the aforementioned approaches considerably reduced protein adsorption and subsequent cell adhesion^34,35^. However, there are a few disadvantages to these approaches, such as complicated processes, use of toxic catalysts on the surface, and expensive equipment and materials. Thus, a coating material that forms an ultrathin layer on fine structures through a simple and biocompatible coating process is desirable. Furthermore, it is beneficial for coated surfaces to tolerate gamma sterilization and are stable in stock and biological environments.

**Figure 1:**
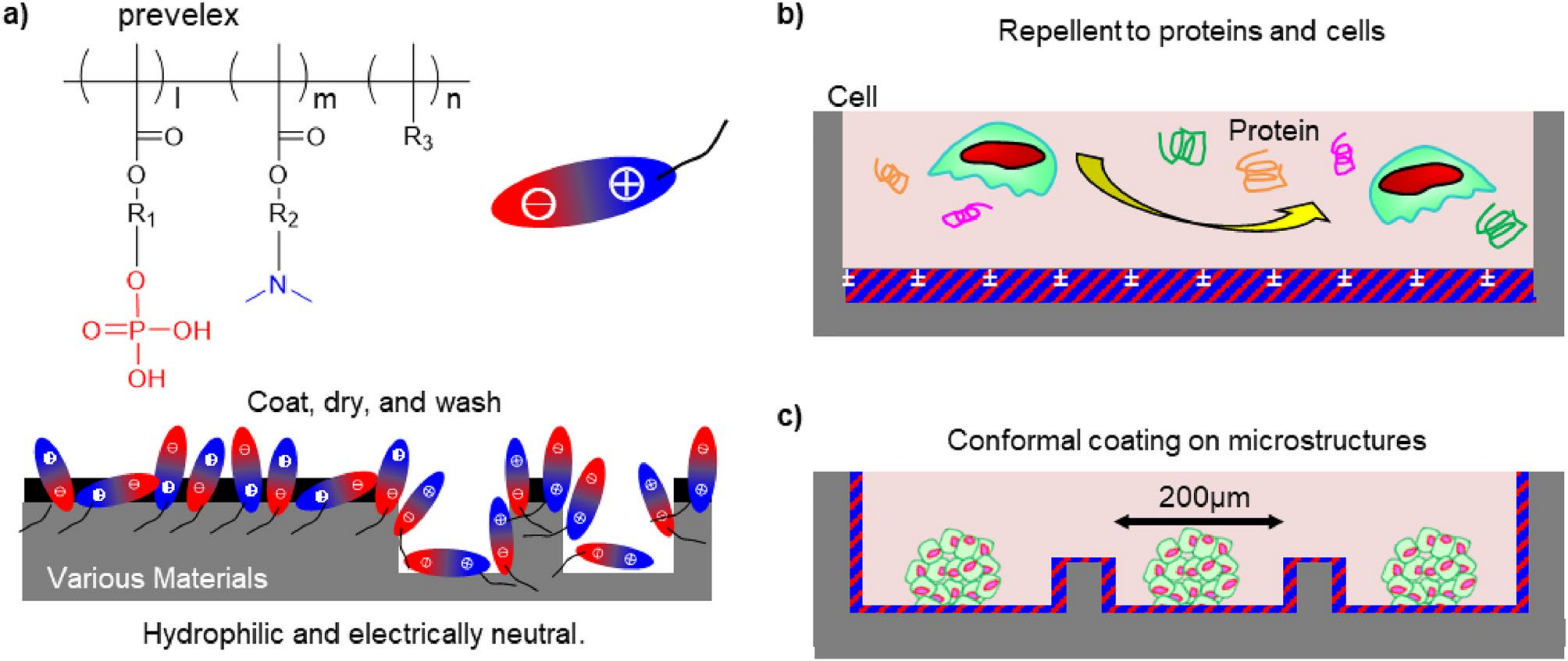
Coating with prevelex and its use for cell aggregate culture. (**a**) Chemical structure of polyampholyte “prevelex” consisting of phosphate units and amine units. Thin polymer films are conformally formed on substrates via simple coating, drying, and washing processes, altering the surfaces to be highly hydrophilic and electrically neutral. (**b**) Prevelex-coated surface for resistance to protein and cell adhesion. (**c**) Spheroids formed in microwell array with conformal prevelex coating.

In this study, we synthesized a polyampholyte based on the MPC polymer framework as a reference because it provides excellent resistance to proteins and cells (Fig. 1). To alleviate the costs of MPC polymers, cationic amine and anionic phosphate units, which are characteristic of MPC, are separated into two monomer units in our polyampholyte ^36^. Prevelex dissolved in an ethanol aqueous solution was dip-coated or spin-casted on a flat surface and micro/nano fabricated surfaces to examine the formation of ultrathin and homogeneous layers. The prevelex layer was physicochemically characterized, and biomolecules and cells were exposed to the surface to examine its antifouling capability. Spontaneous formation of hair follicle germ-like structures was also investigated via prevelex-coated microwell array devices. The polyampholyte can be beneficial for the preparation of robust nonfouling surfaces in biomedical applications.

## Results and discussion

### Surface characterizations of prevelex-coated substrates

The synthesized prevelex was characterized using gel permeation chromatography (GPC Supplementary Fig. S1) and Fourier transform infrared spectroscopy (FT-IR, Supplementary Fig. S2). These spectra revealed that the weight-average molecular weight was ~ 320,000, and the polymer was a copolymer composed of phosphate units and amine units. Prevelex was dissolved in an aqueous ethanol solution and coated onto silicon substrates. X-ray photoelectron spectroscopy (XPS) analysis of the prevelex-coated surface further revealed clear peaks for -NH_4_^+^ (Supplementary Fig. S3), indicating that the amino and phosphate groups were ionically cross-linked to form an ionic complex. Although we performed ^1^H nuclear magnetic resonance spectroscopy, the molecular structure and composition ratio of the polymer could not be determined, most likely because of its complex composition (data not shown).

**Figure 2:**
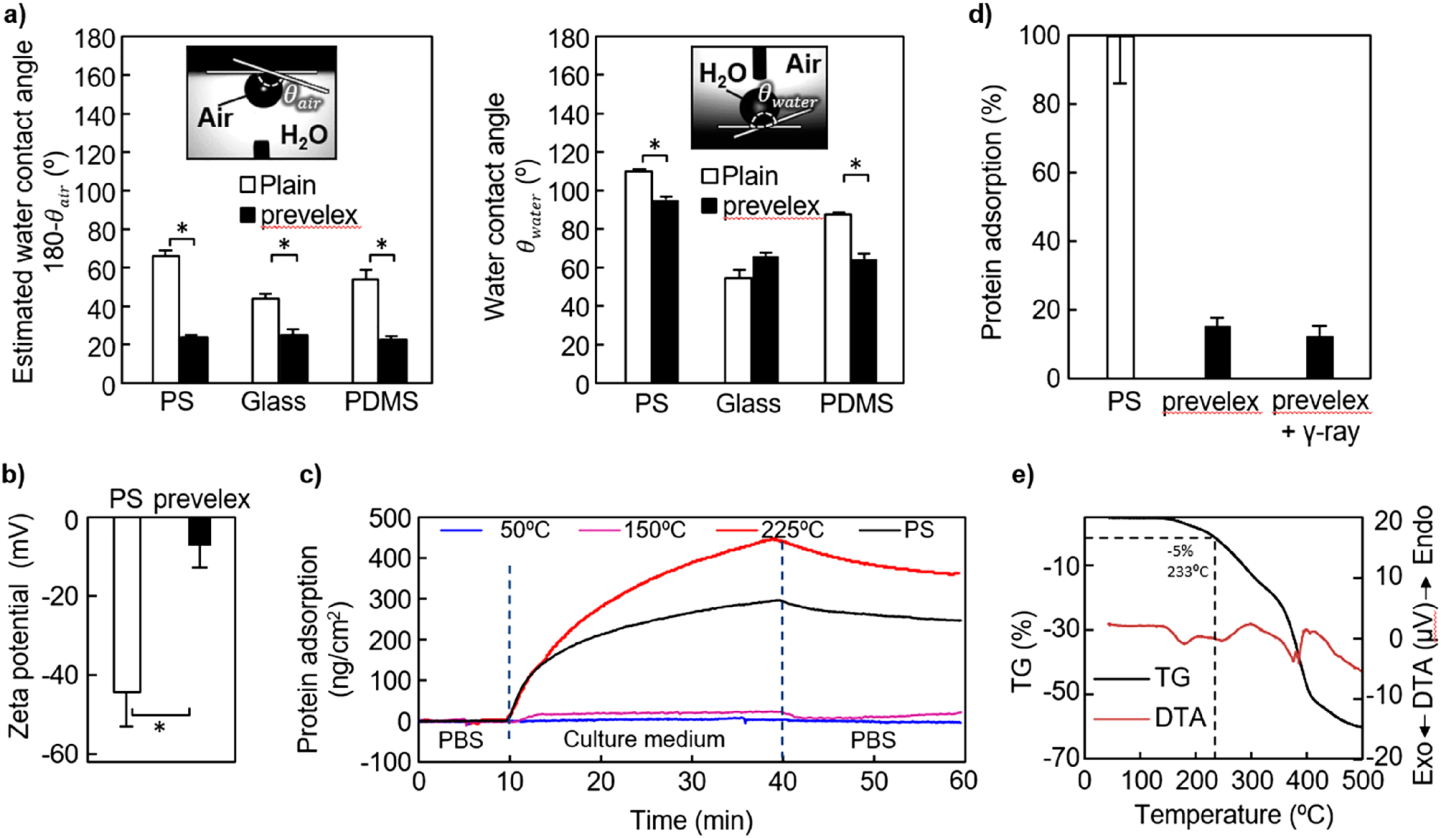
Characterizations of prevelex-coated surfaces. (**a**) Water contact angles with and without prevelex coating on PS, glass, and PDMS substrates under aqueous and dry conditions. The bubble contact angle *θ*_*air*_ is measured with a 2.0 µL air bubble in PBS. The water contact angle *θ*_*water*_ is measured with a 2.0 µL pure water droplet in air. *θ*_*air*_ is converted to *θ*_*water*_ by 180 − *θ*_*air*_. (**b**) Zeta potential of polystyrene substrates with and without prevelex coating. (**c**) Dependence of prebake temperature on protein adsorption. QCM sensor is treated with prevelex and heated at the indicated temperature for 1 h. QCM is used to quantify adsorption of molecules in Eagle’s basal medium supplemented with 10% FBS. (**d**) Protein adsorption after gamma irradiation. The values are presented relative to polystyrene (PS) substrate. (**e**) Thermogravimetric and differential thermal analysis (TG–DTA) of prevelex. Error bars in (**a**), (**b**), and (**d**) represent the standard error calculated from three independent measurements. Numerical variables are statistically evaluated using a student’s t-test, and *p < 0.05 is considered statistically significant.

The wettability and zeta potential are closely associated with the responses of biomolecules and cells on a surface^37^. In this study, prevelex was coated onto polystyrene (PS), glass, and polydimethylsiloxane (PDMS) substrates via a simple dip-coating process, and changes in the wettability and zeta potential were quantified via a static contact angle goniometer and zeta electrometer. As shown in Fig. 2a, the water contact angle under aqueous condition (calculated from the bubble contact angle *θ*_*air*_ by 180 − *θ*_*air*_) significantly decreases to approximately 20° via the prevelex coating irrespective of the original value of the substrates. However, changes in the water contact angles measured under dry condition were inconsistent before and after the coating. This is potentially attributable to the behavior of the polymer side chains consisting of hydrophilic phosphate, and amine units that are sensitive to the wet environment. We considered that the bubble contact angle can be a better indication of non-fouling characteristics because substrates are generally used in aqueous environments in biomedical applications. The zeta potentials of the PS substrates before and after coating are shown in Fig. 2b, where the PS surface was negatively charged. However, the prevelex coating led to an electrically neutral surface. The results suggest that prevelex can be useful for preventing nonspecific adsorption of biomolecules and cells due to the suppression of electrostatic and hydrophobic interactions in liquid/substrate interfaces.

### Stability of the prevelex layer

In the dip-coating processes, prebaking was conducted to evaporate the solvent after casting the prevelex dissolved in an aqueous ethanol solution. Higher temperatures lead to robust film formation and rapid processes, although they can compromise the coating when it exceeds beyond a certain temperature. We examined the effects of prebaking temperature on protein adsorption using a quartz crystal microbalance (QCM). The QCM sensor was coated with prevelex and prebaked at 50 °C, 150 °C, and 225 °C. The surfaces were then exposed to Eagle’s basal medium supplemented with 10% fetal bovine serum (FBS). Figure 2c shows that surfaces treated at 150 °C are resistant to protein adsorption, whereas a significant number of proteins are adsorbed at 225 °C. The results indicated that the prevelex layer tolerates a typical autoclave process for sterilization (121 °C, 20 min). Additionally, we investigated gamma sterilization as it is more often used in commercial culture vessels. The prevelex-coated 96-well plate was exposed to 25 kGy of gamma irradiation (a typical sterilization dose), after which protein adsorption was detected by measuring the enzymatic conversion of the peroxidase substrate. No significant difference in protein adsorption was observed with or without gamma irradiation (Fig. 2d). We used devices after gamma sterilization in subsequent cell culture experiments.

We then performed thermogravimetric/differential thermal analysis (TG–DTA) to examine thermochemical decomposition behavior of the prevelex. TG–DTA was performed under a nitrogen environment. Measurements commenced from 30 to 500 °C at a rate of + 10 °C/min. As shown in Fig. 2e, the weight of the prevelex decreases from 150 °C and significant mass loss is observed at 232 °C (approximately 5.0% loss of the initial mass), and a DTA peak is also observed in the range of 150–190 °C. The results indicate that the prevelex decomposed at temperatures exceeding 150 °C and are consistent with the results in QCM wherein proteins are adsorbed on the surface after prebaking at 225 °C.

### Cell repellent on the prevelex-coated substrate

**Figure 3:**
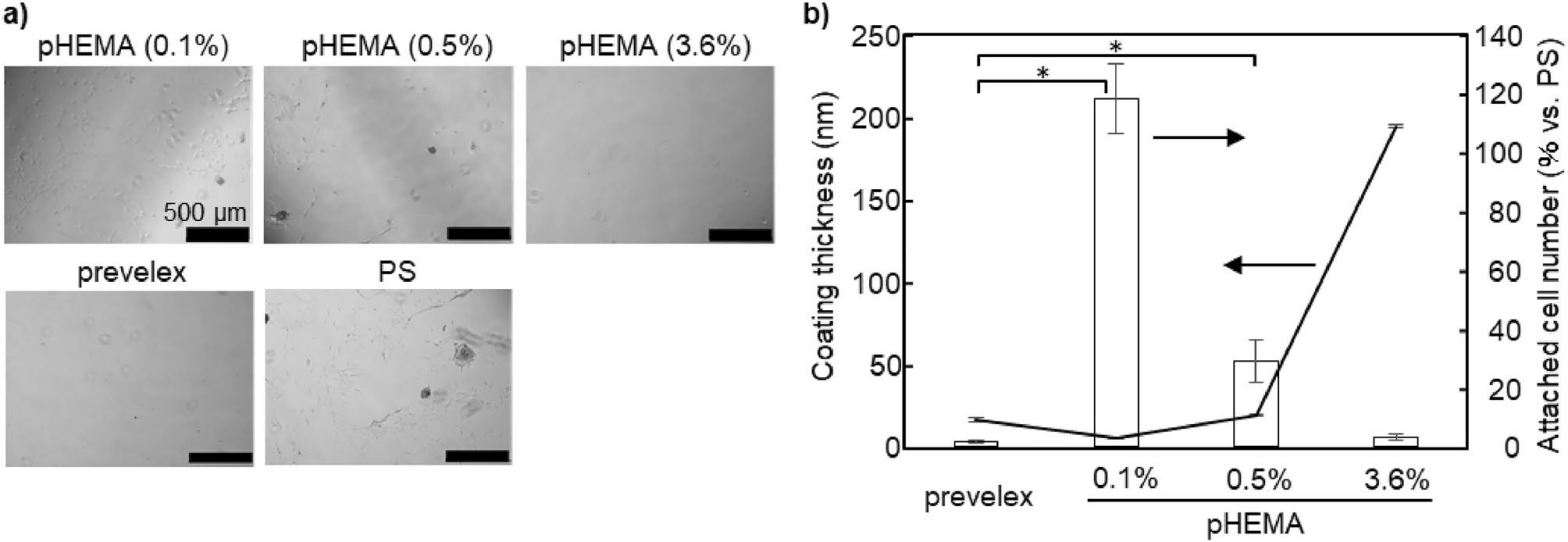
Cell repellent of prevelex-coated surfaces. (**a**) Phase-contrast microscopic images of culture surface 4 days after cell seeding. PS substrates are coated with the different coating agents. (**b**) Coating thickness and attached cell number. Thickness of coating layer was quantified with spectroscopic ellipsometry. Number of attached cells are quantified by measuring the amount of adenosine triphosphate. Error bars represent standard error calculated from three independent measurements. Numerical variables are statistically evaluated using ANOVA, and *p < 0.05 is considered statistically significant.

The resistance of the prevelex-coated surfaces to cell adhesion was examined and compared to that of a typical coating polymer, pHEMA^38^. The polymers were coated onto 24 well PS plates on which 10T1/2 mouse fibroblasts were seeded. The cells attached and spread on a plain PS and 0.1% and 0.5% pHEMA-coated surfaces, whereas only a few cells attached to 3.6% pHEMA- and prevelex-coated surfaces (Fig. 3a). The thickness of the coated layers and number of attached cells were quantified via an ellipsometer and ATP measurements, respectively (Fig. 3b). The thicknesses of the 0.1% and 0.5% pHEMA layers were in the same range as that of the prevelex, although the attached cell number significantly exceeded that of the prevelex. The concentration of pHEMA increased up to 3.6% to almost completely prevent cell adhesion and was comparable to that of the prevelex. However, the layer of 3.6% pHEMA was significantly thicker than that of the prevelex.

### Homogenous coating of prevelex on fine structures

**Figure 4:**
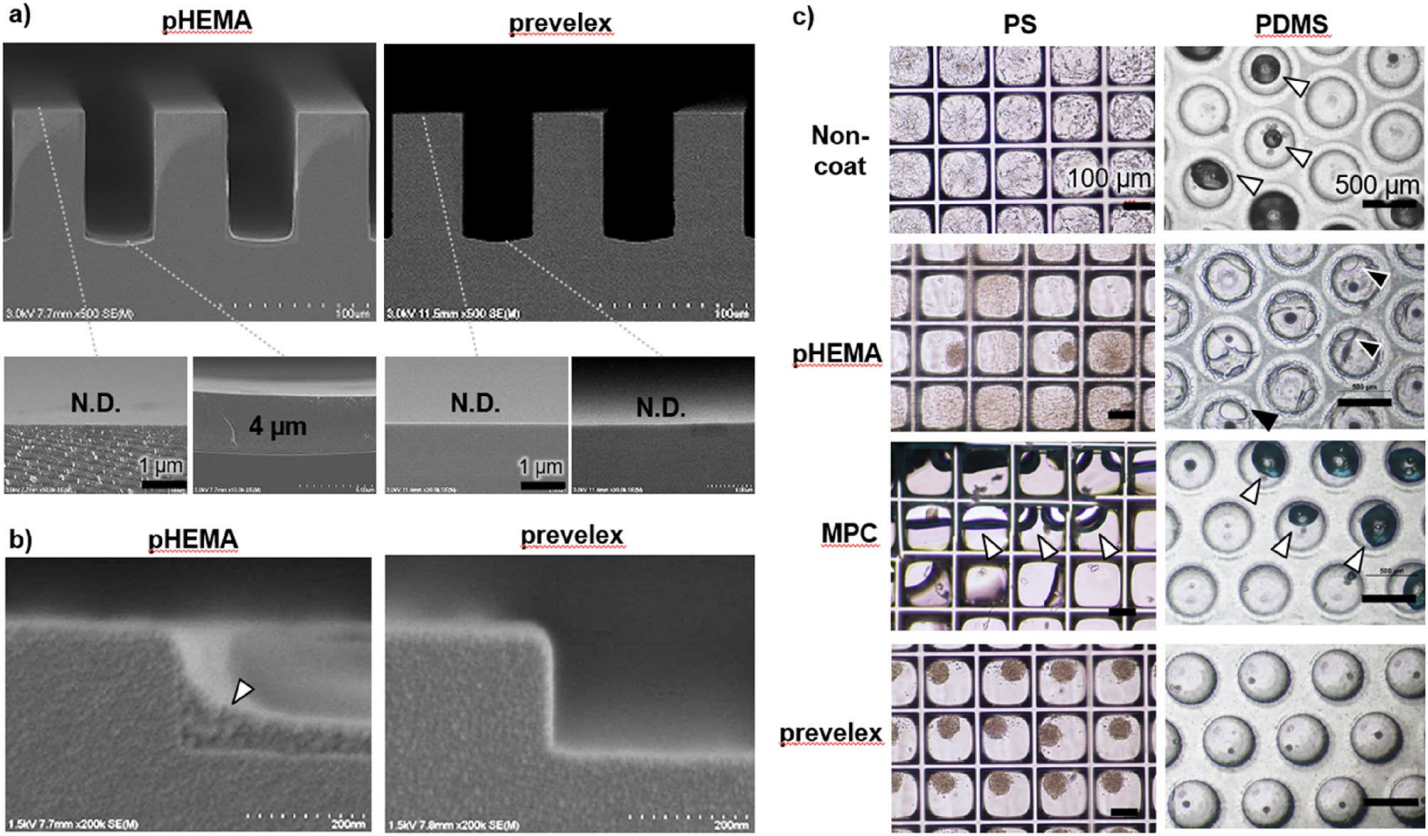
Prevelex coating to microstructures. (**a**) Scanning electron microscopic images of prevelex and 3.6% pHEMA layers coated on microstructured silicon substrates. (**b**) Coating on sub-micrometer stepped surfaces. Height of the step is 200 nm. (**c**) Comparisons of coating agents on microwell array plates. PS and PDMS plates are coated with three different coating agents. Cells are observed four days after seeding. White and black arrowheads indicate air bubbles and deformed layers of coating resin, respectively.

To examine the coating characteristics of fine structures, 3.6% pHEMA and prevelex were coated on micro/nano-fabricated silicon substrates, which were then cross-sectioned and observed via a scanning electron microscope (Fig. 4a). The thickness of the pHEMA layer was significantly different based on the location on the concave–convex surface, which was approximately 4 µm on the bottom surface and less than the limit (< 10 nm) on the top surface. Prevelex conduced to a uniform and ultra-thin layer (< 10 nm) on the top and bottom surfaces. Furthermore, pHEMA formed a thick layer on the corners of the steps in a relatively shallow concavo–convex surface, whereas the prevelex formed a conformal and ultra-thin layer even in such portions (Fig. 4b). To quantify the thickness of the prevelex layer, edges of the prevelex layer on a flat silicon substrate were scanned using an atomic force microscopy (Supplementary Fig. S4). The thickness of the prevelex layer was 6.6 nm, and the roughness over a scanning distance of 10 μm was 0.26 nm. The polymer thickness directly exerted cell-repellent effects on pHEMA. Thus, non-uniform layers can compromise cell-repellent characteristics. The thin and conformal coating of the prevelex can be attributed to electrostatic interactions between the polymer molecules and between the polymer molecules and surface. Prevelex is composed of hydrophilic anionic phosphate units, cationic dimethyl amino units, and a hydrophobic alkyl backbone (Fig. 1a). Therefore, it electrostatically adsorbs onto charged surfaces such as PS (Fig. 2b). Stacking of additional layers of prevelex can occur until the surface net charge is sufficiently neutralized, leading to an ultra-thin and homogeneous layer on the micro-nano structures. Conversely, pHEMA forms thick layers in the coating and drying processes through intermolecular hydrogen bonding between the hydroxyl group, which retains the thick layer even after washing processes. The dip-coated MPC polymer layer has been reported to be ~ 50 nm^39^. The difference in the thickness between the prevelex and MPC polymer layers may be because the MPC polymer contains ~ 70% hydrophobic units (butyl methacrylate) whereas prevelex does not.

### Cell-repellent coating on microfabricated cell culture devices

Three-dimensional cell aggregate culture, including spheroid and organoid culture, has been typically conducted using non-cell adhesive microfabricated devices^4^. A PS device with flat square bottom microwell arrays and a PDMS device with U-shaped round bottom microwell arrays were coated with three cell-repellent coatings as follows: 3.6% pHEMA, MPC polymers, and prevelex. These surfaces were washed with PBS and incubated in biological environments in culture medium. The pH of the washing solution and culture medium were in the range of 7.1–7.7 and 7.0–7.4, respectively. Additionally, the ionic strength of the washing solution and culture medium were determined to be 13.1 and 12.2 mS/cm, respectively, using an electrical conductivity meter (CM-42X, DKK TOA, Tokyo, Japan). A suspension of 10T1/2 mouse fibroblasts was then poured on the coated devices. Figure 4c shows phase-contrast microscopic images after one day of culture. Without coating, the cells attached and spread on both devices, and air bubbles remained in some microwells in the PDMS device. Spheroid formation was observed on the PS device coated with pHEMA. However, in most microwells, the cells attached and spread over the entire surface. On the PDMS device coated with pHEMA, the coated polymer resin was deformed into heterogeneous layers, and this was potentially attributable to swelling and relatively weak adhesion of the layers on the surface under cell culture conditions. When coated with MPC polymers, air bubbles remained in almost all microwells in the PS and PDMS devices. This is potentially because MPC polymers possess approximately 70% hydrophobic butyl methacrylate units, which are moderately hydrophobic under dry conditions^40^. The air bubbles disappeared when coated with prevelex, and the cells formed spheroids in all the microwells in the PS and PDMS devices.

### Spheroid formation of various types of cells on prevelex-coated microwell array devices

**Figure 5:**
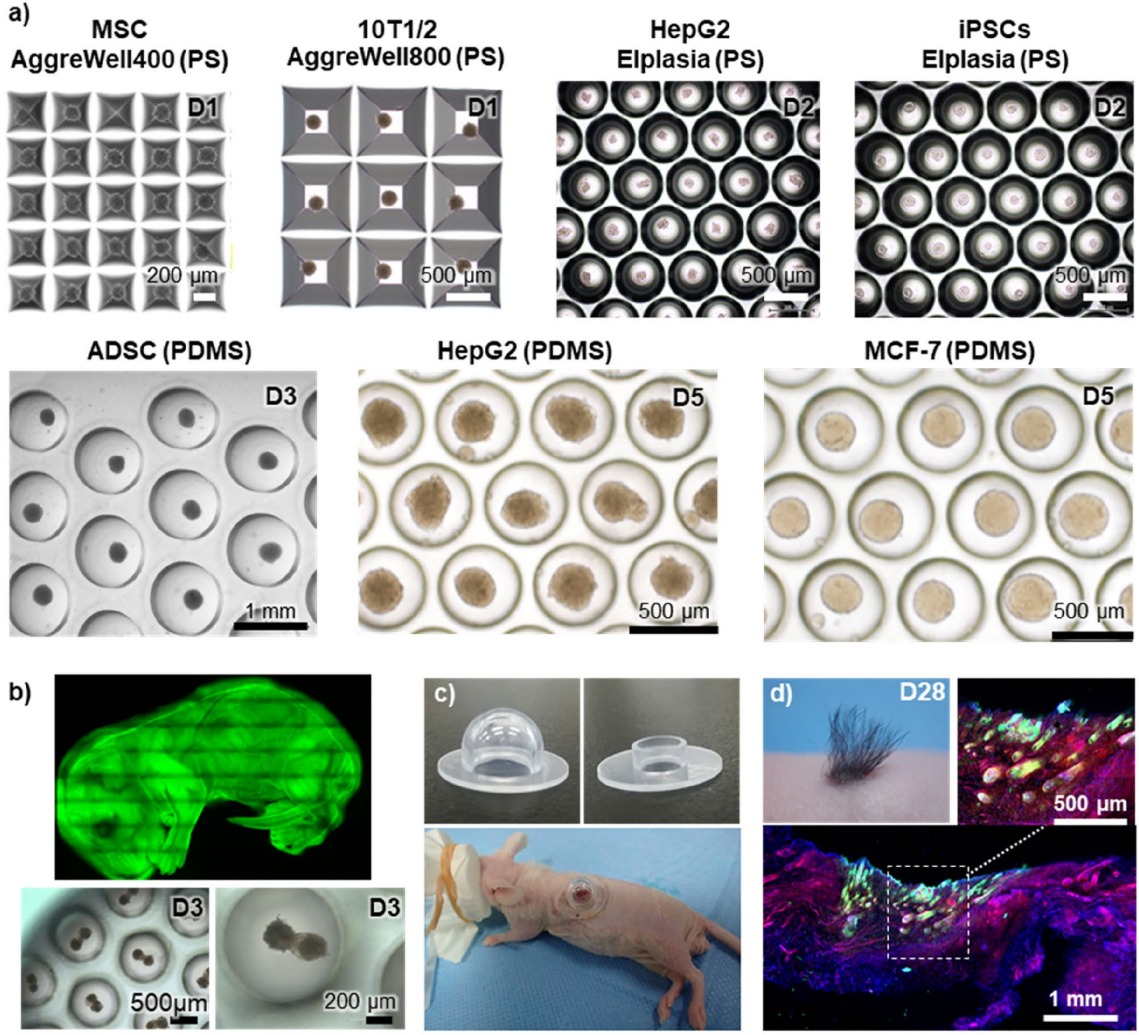
Aggregate formation of various cell types on prevelex-coated microwell array plates and hair follicle neogenesis upon transplantation of aggregates of GFP-labeled cells. (**a**) Spherical aggregate formation. PS and PDMS plates with square pyramid and hemispherical microwell arrays are coated with prevelex, and the cells indicated are seed and cultured for 1–5 days. (**b**) Transgenic fetus mice expressing green fluorescence protein (GFP) and hair follicle germ-like aggregates formed at three days of culture. Embryonic epithelial and mesenchymal cells are isolated from the fetus and let them form aggregates in the prevelex-coated PDMS microwell array plate. (**c**) Lab-made chambers for patch assay with nude mice. (**d**) Hair follicle neogenesis in vivo. The aggregates composed of the two cell types are transplanted into the dorsal skin of nude mice. Appearance of generated hair shafts is shown. The fluorescent images are from histological sectioning of the transplanted site. Rhodamine-phalloidin and DAPI staining were used to visualize actin cytoskeleton and nuclei of all the cells in the skin sections. GFP indicates cells from grafts.

To examine the robust and versatile use of prevelex, different types of cells were seeded into different prevelex-coated microwell array devices. The cell types included human mesenchymal cells (MSCs), 10T1/2 mouse fibroblasts (10T1/2), human hepatoblastoma cells (HepG2), human induced pluripotent stem cells (iPSC), human adipose stem cells (ADSCs), and human breast cancer cells (MCF-7). Spheroid formation in each microwell was observed with all combinations of cell types and the microwell array devices used in the study (Fig. 5a).

In contrast to devices made of PS and other plastic resins, a PDMS plate is oxygen-permeable, which enables the supply of oxygen through the bottom of the microwells. This is crucial when cells form three-dimensional aggregates at a high cell density. We previously reported that a PDMS microwell array device was useful for large-scale preparation of hair follicle germ-like aggregates as tissue grafts for hair regenerative medicine, whereas the same microwell array device composed of PS did not show hair follicle germ formation^35,41^. In previous studies, the PDMS microwell array device was modified with a block copolymer comprising poly(oxyethylene) and poly(oxypropylene) segments to prevent cell adhesion^42,43^. However, the molecule is a non-ionic surfactant, and severe cytotoxicity was observed unless substantial washing was performed after coating to remove excess polymers. Additionally, because of instability of the coated layer, neither autoclave nor gamma sterilization was applicable to coated devices. Thus, a coating solution should be sterilized by filtration and then coated to autoclaved devices immediately before the use. In this study, the prevelex was non-cytotoxic in culture, and the device was sterilized with gamma rays and stored in a plastic bag as ready-to-use. We expected that the ultrathin layer of the prevelex does not hinder the oxygen permeability of PDMS. To distinguish cells in grafts from recipient tissues, cells were isolated from transgenic fetuses expressing green fluorescence protein (GFP) (Fig. 5b). A mixed cell suspension of GFP-labeled embryonic epithelial and mesenchymal cells was poured into the prevelex-coated PDMS microwell array device. The cells formed aggregates in each well after one day of culture in which the two types of cells were randomly distributed. During the three days of culture, the two cell types were spatially separated from each other and exhibited typical morphological features of hair follicle germs observed during in vivo development (Fig. 5b). Subsequently, 250 aggregates were transplanted into a surgically generated wound with silicone chambers on the dorsal skin of mice (Fig. 5c). Vigorous de novo hair generation was observed at transplantation sites 28 days after transplantation (Fig. 5d). Confocal laser scanning microscopy revealed that GFP-labeled cells constituted newly formed hair follicles. In the animal experiments, the aggregates were collected and transplanted on the third day of culturing. However, the aggregates were maintained in vitro even when the culture continued for 15 days (Supplementary Fig. S5), confirming the long-term stability of the prevelex-coated surface.

## Conclusions

In the study, we demonstrated that the polyampholyte coating material, prevelex, provides a hydrophilic and electrically neutral film on substrates. The coating of the prevelex can be performed via dip-coating, thereby forming ultrathin (approximately 20 nm) and robust nonfouling layers on the PS and PDMS substrates. The characteristics of the material, such as the relatively low prebake temperature (50–150 °C) and conformal coating, illustrate that the coating process was straightforward and preferable for various micro/nano-fabricated devices. The microwell array devices coated with prevelex successfully induced spheroid formation in various cell types, whereas those coated with pHEMA and MPC polymers were problematic due to air bubble residue in the microwells and/or deformation of coated polymer films. To examine cellular functionalities, a mixture of mouse embryonic epithelial and mesenchymal cells was seeded into a prevelex-coated microwell array device. The two types of cells formed hair follicle germ-like aggregates in the device, which generated de novo hair follicles in the dorsal skin of nude mice when transplanted.

Evidently, further investigations should be performed to examine the long-term stability of the coated layer and maintenance of cell-repellent capability against spheroids and other tissue grafts. Notably, a prevelex-coated PS microwell array device is currently being used in clinical trials by another research group in Japan (details of which cannot be disclosed due to confidentiality agreements). Our next step involves conducting comprehensive studies to further elucidate underlying mechanisms for nonfouling coating with prevelex and characterize dynamics of polymer release on various material surfaces. The coating material is expected to provide a robust and fine coating approach for preparation of non-fouling substrates for biomedical applications.

## Materials and methods

### Synthesis and characterization of prevelex

Prevelex was synthesized as previously reported^34^ and characterized using GPC and FT-IR spectroscopy to determine the molecular weight and functional groups. Subsequently, prevelex was dissolved in an aqueous ethanol solution and coated onto silicon substrates. The prevelex-coated silicon substrate was analyzed using XPS to determine the functional groups on the surface.

### Wettability changes

PDMS plates were prepared via mixing the prepolymer solution and curing agent at a ratio of 10:1 and then baking at 100 °C for 1 h (SYLGARD™184 Silicone Elastomer Kit, Toray Daw Corning, Japan). The surfaces of PS (Asnol Petri Dish, AS ONE Corporation, Japan), glass (D263Teco, AS ONE Corporation, Japan), and PDMS plates were cleaned with oxygen etching (5 mA, 3 min) (SEDE-GE, Meiwa Fosis, Japan) and immersed in the prevelex solution for 10 s. This was followed by solvent evaporation at 50 °C for 24 h under atmospheric conditions. The plates were then washed with pure water thrice for 10 s to remove excess polymers.

The changes in the wettability of surfaces via prevelex coating were quantified via air and water contact angles using a static contact angle goniometer (DMC-MC3, Kyowa Interface Science Co., Ltd, Tokyo, Japan). The bubble contact angle *θ*_*air*_ was measured by attaching sample plates to a custom holder filled with PBS. An air bubble (2.0 μL) was introduced through a U-shaped needle, and the contact angle *θ*_*air*_ was measured via photographic images. To measure the water contact angles *θ*_*water*_ under dry conditions, a pure water droplet (2.0 μL) was placed onto sample plates and contact angles *θ*_*water*_ were measured within 1 s using photographic images. Data were collected from four independent experiments using each sample plate.

### Zeta potential measurement

The PS plate (1 mm thick) was coated with prevelex as aforementioned and cut into 1 mm × 1 mm sections. The plate was attached to a stage for a zeta potential measurement unit (ZEN1020, Malvern). The unit was set into a measurement cell (10 × 10 × 45 mm). The surface zeta potential was measured in 100X-diluted D-PBS(-) using Zetasizer Nano (Malvern). Polymer latex (micromer 1 μm, micromod) was used as the zeta potential transfer standard. A PS plate without a coating was used as a control. Data were collected from four independent experiments.

### Protein adsorption measurement

The thermal stability of the prevelex layer was evaluated using a QCM. QCM-Au sensors (Biolin Scientific, Sweden) were cleaned via oxygen etching (5 mA, 3 min) and precoated with a polystyrene solution (331651-25G, Sigma-Aldrich) in 1% toluene (50070-0330, Junsei Chemical Co.). The prevelex solution was coated on sensors via spin-coating (3500 rpm, 30 s) using a spin coater (Opticoat MS-B100, MIKASA, Japan). The sensors were then baked at temperatures ranging from 50 to 225 °C for 1 h. After rinsing in PBS with ultrasonication for 5 min, the sensors were placed in the chamber of a QCM equipment (QSense, Biolin Scientific, Sweden). Subsequently, PBS was introduced through tubing for 10 min and was followed by Eagle’s basal medium supplemented with 10% fetal bovine serum (Sigma-Aldrich) for 30 min. This was followed by 20 min of PBS for rinsing. The flow rate was 50 μL/min. The amount of protein adsorption was calculated via the Sauerbrey equation. Δm = − C·Δf/n (C = 17.7 ng/Hz/cm^2^, n = 9). Specifically, Δf was calculated as the change in frequency at 10 min and 60 min. Data were collected from three independent experiments.

The influence of gamma irradiation on the prevelex layer was evaluated using enzyme proteins. Prevelex was coated onto a 96-well culture plate (Falcon #351172, Corning, New York, USA). The prevelex solution was poured into the wells, the excess solution was aspirated, and the solvent was evaporated at 50 °C for 24 h. The plates were then washed with pure water thrice for 10 s. The plate was exposed to 25 kGy of gamma irradiation. Horseradish peroxidase-conjugated immunoglobulin (Proteintech, Illinois, USA) in 100 µL PBS was added to each well and incubated for 30 min. After aspirating the enzyme solution and washing with PBS thrice, 100 µL of tetramethylbenzidine peroxidase substrate solution (SureBlue, SeraCare Life Sciences, Massachusetts, USA) was added. The reactions were terminated after 60 s by adding 100 µL of stop solution (SureBlue, SeraCare Life Sciences, Massachusetts, USA). The absorbance at 450 nm was measured using a plate spectrophotometer (Infinite 200 PRO, TECAN, Zurich, Switzerland) to estimate the amount of protein adsorbed.

### Thermogravimetric and differential thermal analysis (TG–DTA)

The prevelex solution was evaporated using a rotary evaporator (EYELA, Japan) at 40 °C for 3 h to remove the solvent completely, and the polymer solid (4.5 mg) was placed on an aluminum pan. The TG–DTA was performed using a TG–DTA apparatus (RG8120, Rigaku, Japan) under a nitrogen environment. The temperature increased from 30 to 500 °C at a rate of + 10 °C/min. Alumina was used as a reference.

### Thickness of coated polymer layers

Highly polished silicon wafers (GlobalWafers Co., Taiwan) were modified with hexamethyldisilazane (HMDS) at 90 °C for 30 s (ACT 8, Tokyo Electron, Japan).

p(HEMA) (P3932-25G, Sigma-Aldrich) was dissolved in 95% ethanol solution, and 0.1%, 0.5%, and 3.6% p(HEMA) solutions were prepared. The p(HEMA) and prevelex solutions were spin-coated (1500 rpm, 60 s) on the HMDS-modified silicon wafers. The wafers were then baked at 50 °C for 24 h, rinsed with pure water, and dried at 50 °C for 1 h. The dry thickness of the polymer layers was quantified using spectroscopic ellipsometry (M-2000, J. A. Woolam Co., USA). Thickness was calculated using Cauchy’s equation.

$${\text{n}} = {\text{A}} + {\text{B/}}\lambda^{{2}} + {\text{C/}}\lambda^{{4}} + \cdots$$

Data were collected from three independent experiments.

### Cell adhesion on polymer-coated flat surfaces

The prevelex and p(HEMA) solutions were dispensed into 24 well tissue culture plates (SUMILON #MS-80240, Sumitomo Bakelite Co., Japan). After 1 h, the coating solutions were aspirated, and the culture plates were baked at 50 °C for 24 h and rinsed with pure water. Additionally, C3H10T1/2 mouse fibroblasts (DS Pharma Promo Co., Japan) were maintained in Eagle’s basal medium (Thermo Fisher Scientific, USA) supplemented with 10% fetal bovine serum and 1% penicillin–streptomycin-glutamine (Thermo Fisher Scientific, USA) at 37 °C under a 5% CO_2_ environment. Cells at three days of culture were harvested with 0.25% trypsin for 5 min at 37 °C and were seeded on the polymer-coated 24 well plates at 8 × 10^4^ cells/well.

After four days of culture, the medium was aspirated, and each well was rinsed with 500 μL of the culture medium thrice to remove non-adherent cells. Images of attached cells were captured via a phase-contrast microscope (Eclipse TS100, Nikon, Japan). To quantify the number of attached cells, an adenosine triphosphate (ATP) assay was conducted based on the manufacturer’s protocol (Cell Titer-Glo, Promega Co., USA). The absorbance at 450 nm was measured via a plate reader (Infinite M200PRO, Tecan).

### Polymer coating on fine silicon structures

Two types of micro/nanopatterned silicon substrates were fabricated by etching two line-patterns (width/height = 50 μm/80 μm and 50 μm/230 nm). The substrates were then modified with HMDS to make the surface hydrophobic. The prevelex and 3.6% p(HEMA) in 95% ethanol solution were coated onto the HMDS-modified silicon substrates. After deposition of platinum, cross-sectional images were obtained with a scanning electron microscope (S-4800, Hitachi High-Tech Co., Japan) at an acceleration voltage of 1.5 kV.

### Spheroid formation test on micropatterned cell culture devices

In addition, MPC polymers were synthesized as previously reported^18^. After cleaning with oxygen etching, prevelex, 3.6% p(HEMA), and 0.5% MPC polymers were coated onto the plain PS plate with square microwells (Elplasia, Kuraray Co., Ltd, Japan) and a PDMS plate with U-shaped microwells. A suspension of 10T1/2 fibroblasts in Eagle’s basal medium was seeded into the PS (0.2 × 10^4^ cells/well) and PDMS (30 × 10^4^ cells/plate) plates. The cells were then cultured for four days to examine spheroid formation.

To investigate the robust and versatile usage of prevelex on different culture plates and cell types, seven types of cells were seeded on five types of micropatterned plain plates that were cast coated with prevelex prior to their use. The combinations of culture plates, cell types, and cell seeding density were as follows: Aggrewell 400, 800 (Stemcell Technologies, Canada), MSCs (Promocell, Germany) and 10T1/2 fibroblasts at 60 × 10^4^ cells/well; Elplasia (Kuralay Co., Ltd), HepG2 (DS Pharma Biomedical, Japan), and human iPSC (Center for iPS Cell Research and Application, Japan) at 1.0 × 10^4^ cells/well; lab-made PDMS plates with microwells with 500 μm in diameter, ADSCs at 10 × 10^4^ cells/well; lab-made PDMS plates with microwells with 1000 μm in diameter; and HepG2 and MCF-7 at 30 × 10^4^ cells/well.

### Animals

Pregnant C57BL/6 mice and C57BL/6-Tg (CAG-EGFP) mice were purchased from CLEA (Japan) and SLC (Japan), respectively. Five-week-old ICR nu/nu mice were purchased from Charles River, Japan. The animal study was approved by the Committee on Animal Care and Use, Yokohama National University (Permit numbers: 2019-04 and 2019-06). The care and handling of mice conformed to the requirements of the Animal Care and Use Committee of Yokohama National University.

### Preparation of mouse epithelial and mesenchymal cells^28^

Embryonic mice (E18) were extracted from a C57BL/6 or C57BL/6-Tg (CAG-EGFP) pregnant mice, and small pieces of their back skin were harvested. After aseptic treatment with 4.8 U/ml dispase II (Sigma Aldrich, St. Louis, MO, USA) for 60 min, the epithelial and mesenchymal layers were separated using tweezers. The epithelial layer was then treated with 100 U/ml collagenase type I (FUJIFILM Wako Pure Chemical Corporation, Japan) for 80 min and 0.25% trypsin for 10 min at 37 °C. The dermal layer was treated with 100 U/ml collagenase type I for 80 min at 37 °C. Debris and undissociated tissues were removed using a 40 µm mesh cell strainer. After centrifugation at 200 × *g* for 3 min, epithelial and mesenchymal cells were resuspended in KG2 (Kurabo, Osaka, Japan) and DMEM (Sigma Aldrich), respectively. Freshly isolated cells were used for the experiments without passaging in culture. When the cells were mixed for co-culture, we used a mixed culture medium of DMEM and KG2 at a 1:1 ratio supplemented with 10% FBS (Sigma Aldrich) and 1% penicillin–streptomycin (Thermo Fisher Scientific, Waltham, MA, USA).

### Preparation of HFG-like aggregates

Epithelial and mesenchymal cells at the same density were seeded into the PDMS plate and cultured to fabricate HFGs, as previously reported^35^. Cell suspensions (2 mL) containing epithelial and mesenchymal cells (total cell density 1 × 10^6^ cells/mL, 1:1 ratio) were poured into a PDMS plate and cultured in a mixed culture medium of DMEM and KG2 for 3 days. The self-organization of the two cell types in the HFGs was examined after three days using a fluorescence microscope (BZ-X810, Keyence, Japan).

### Hair-chamber assay

The hair-induction ability of HFGs was quantified using a hair-chamber assay as described previously^41^. Under isoflurane anesthesia, a full-thickness wound (4–6 mm in diameter) was surgically generated on the back skin of five-week-old ICR nu/nu mice. The HFGs prepared using GFP-labeled epithelial and mesenchymal cells dissociated from C57BL/6-Tg (CAG-EGFP) mice were transplanted at a density of 250 aggregates/chamber into a silicone chamber (Nissan Chemical Industries, Tokyo, Japan) inserted, and sewn onto the muscle fascia. The upper chamber was removed a week after the transplantation, the lower chamber was removed after a week, and hair growth was monitored for 4 weeks. All transplanted sites were observed via a digital camera (Tough, Olympus, Japan). The transplanted skin was cut into small pieces and fixed 4 weeks after transplantation with 4% paraformaldehyde (FUJIFILM Wako Pure Chemical Corporation). A small piece of the skin was stained with rhodamine-phalloidin (Cytoskeleton, Denver, CO, USA) and DAPI (Sigma Aldrich) and observed using a confocal laser microscope (LSM 700; Carl Zeiss).

### Statistical analysis

Data for each experiment are presented as the mean ± standard deviation of at least three independent experiments. Statistical evaluation of numerical variables was conducted using Student’s t-test, and differences with *p* values of less than 0.05, were considered statistically significant.

## Supplementary Information

Supplementary Figures.
