# Supplementary material for: Cell-repellent polyampholyte for conformal coating on microstructures
Source: Sci Rep. 2022 Jun 25;12:10815. doi: 10.1038/s41598-022-15177-8 (PMC9233689; doi:10.1038/s41598-022-15177-8)
Supplement: Supplementary file 1 — Supplementary Figures. [file 41598_2022_15177_MOESM1_ESM.docx]

**Supporting Information for:**

Cell-repellent polyampholyte for conformal coating on microstructures

Kohei Suzuki Yoshiomi Hiroi, Natsuki Abe-Fukasawa, Taito Nishino, Takeaki Shouji, Junko Katayama, Tatsuto Kageyama, and Junji Fukuda*

*SI correspondence to:

Junji Fukuda: fukuda@ynu.ac.jp


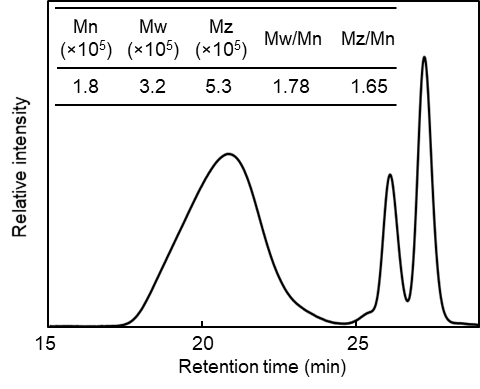


**Fig. S1.** **Gel permeation chromatography (GPC) spectrum.**

Prevelex was analyzed using GPC equipped with a refractive index detector (Prominence, Shimadzu, Kyoto, Japan). Polymer solutions (1 mg/ml, in ionic aqueous solution) were injected and eluted at a flow rate of 1 ml/min. The calibration curve was obtained using a set of primary polyethylene oxide standards. The weight-average (Mw), number-average (Mn), and z-average (Mz) molecular weights were calculated from the spectrum.


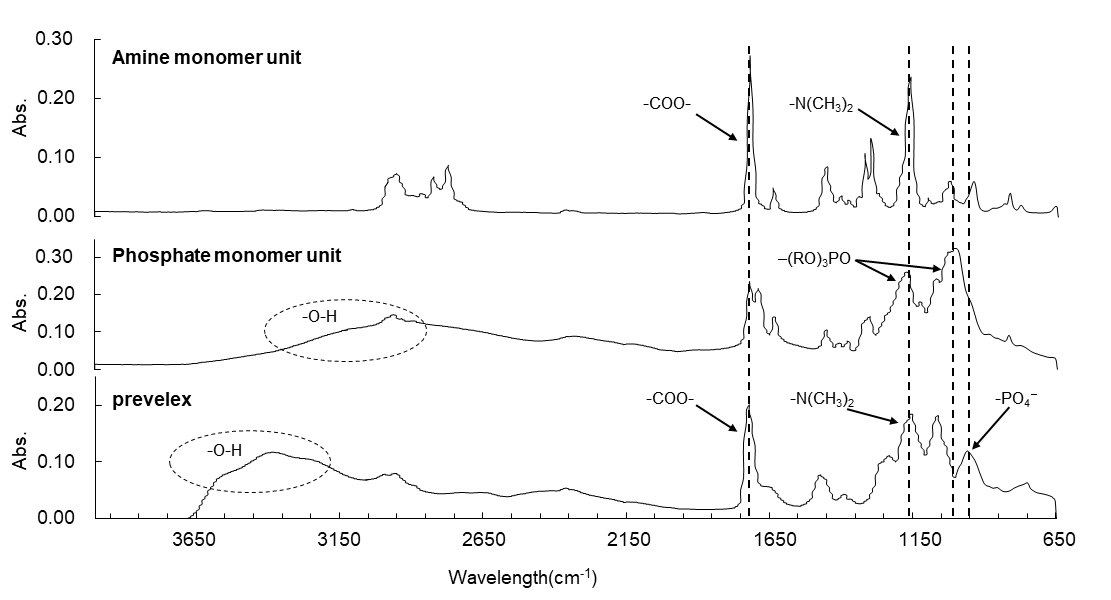


**Fig. S2.** **Fourier transform infrared (FT-IR) spectra.**

The chemical structures of each monomer and prevelex were analyzed by FT-IR (Nicolet iS5 with ATR iD5, Thermo Scientific, Massachusetts, USA) in the 4000–400 cm^−1^ range, at a resolution of 4 cm^−1^. The characteristic peaks are attributed to –O–H (3000~3500cm^−1^), –C=O (1750 cm^−1^), –N(CH_3_)_2_ (1160 cm^−1^) , –(RO)_3_PO (1000 and 1160 cm ^−1^), and –PO4^-^ (960 cm ^−1^) stretching vibrations.


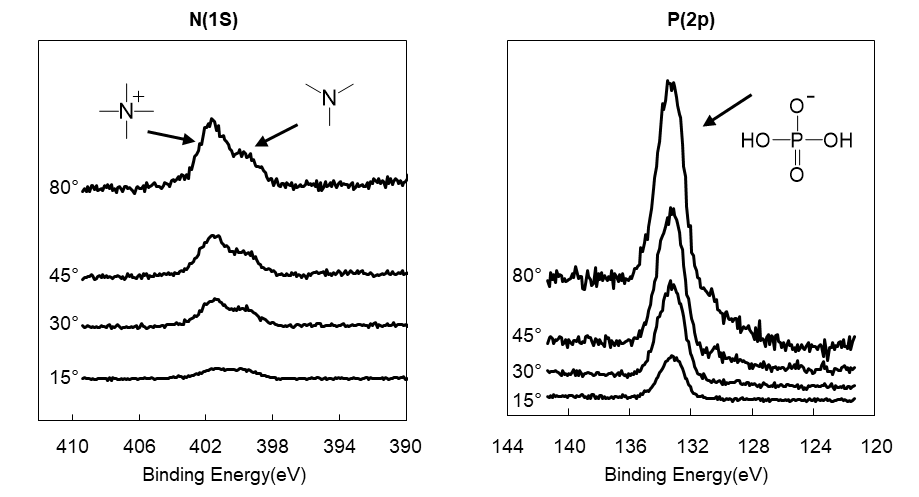


**Fig. S3. X-ray photoelectron spectroscopy (XPS) spectra of a prevelex-coated silicon substrate surface.**

The chemical structure of prevelex on the surface was analyzed by XPS (PHI Quantera SXM, ULVAC-PHI, Kanagawa, Japan) with Al Kα X-rays (1486.6 eV). During the analyses, the pressure in the chamber was set to be ~10^−9^ Torr. The measurements were carried out at 15, 30, 45, and 80° photoelectron take-off angles relative to the surface with an acceptance angle of ± 3°. The analyzer pass energy was set at 23.555 eV for the high-resolution spectra.


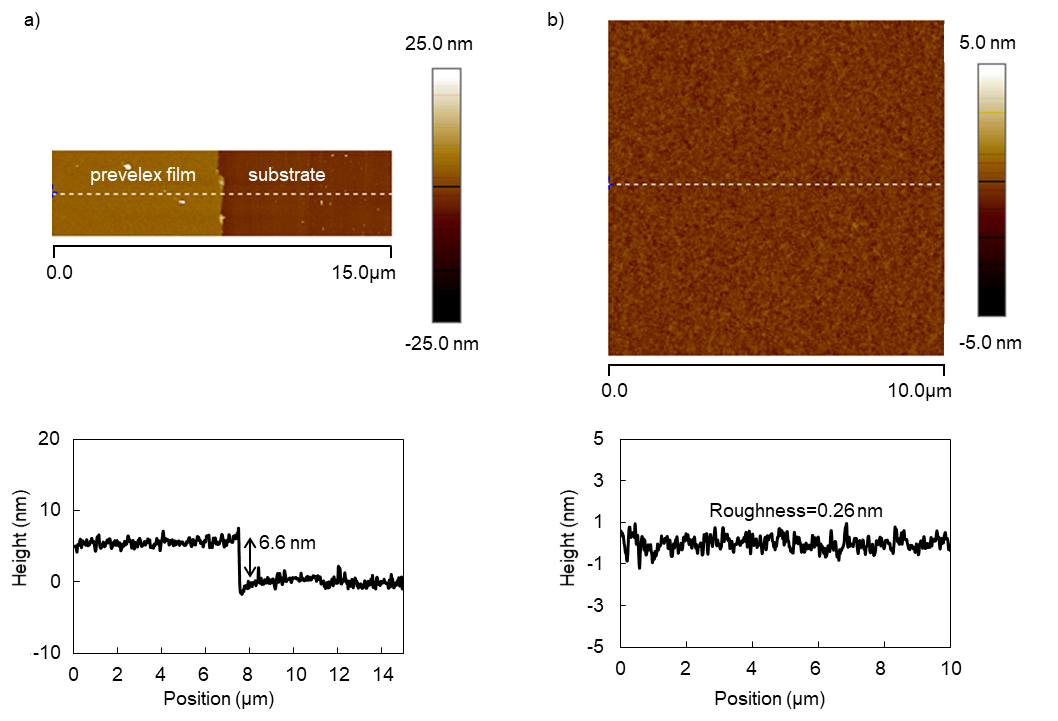


**Fig. S4. Atomic force microscopy images of prevelex-coated silicon substrates.**

a) The film thickness of prevelex on silicon substrates and b) the roughness of the surface. The measurements were conducted using atomic force microscopy (Dimention Icon, Bruker, MA, USA) with a tip (RTESPA-150, Bruker) in tapping mode at a frequency of a) 0.4 Hz and b) 0.8 Hz.

**Fig. S5. Long-term culture of aggregates in prevelex-coated microwells.**

Epithelial and mesenchymal cells were mixed and seeded in the prevelex-coated microwell array plate and cultured for 15 days (D1–15).
